# Mechanotransduction of matrix stiffness in regulation of focal adhesion size and number: reciprocal regulation of caveolin-1 and β1 integrin

**DOI:** 10.1038/s41598-017-14932-6

**Published:** 2017-11-08

**Authors:** Yi-Chun Yeh, Jin-Ying Ling, Wan-Chun Chen, Hsi-Hui Lin, Ming-Jer Tang

**Affiliations:** Department of Physiology, National Cheng Kung University, Tainan, Taiwan

## Abstract

Focal adhesion (FA) assembly, mediated by integrin activation, responds to matrix stiffness; however, the underlying mechanisms are unclear. Here, we showed that β1 integrin and caveolin-1 (Cav1) levels were decreased with declining matrix stiffness. Soft matrix selectively downregulated β1 integrin by endocytosis and subsequent lysosomal degradation. Disruption of lipid rafts with methyl-β-cyclodextrin or nystatin, or knockdown of Cav1 by siRNA decreased cell spreading, FA assembly, and β1 integrin protein levels in cells cultured on stiff matrix. Overexpression of Cav1, particularly the phospho-mimetic mutant Cav1-Y14D, averted soft matrix-induced decreases in β1 integrin protein levels, cell spreading, and FA assembly in NMuMG cells. Interestingly, overexpression of an auto-clustering β1 integrin hindered soft matrix-induced reduction of Cav1 and cell spreading, which suggests a reciprocal regulation between β1 integrin and Cav1. Finally, co-expression of this auto-clustering β1 integrin and Cav1-Y14D synergistically enhanced cell spreading, and FA assembly in HEK293T cells cultured on either stiff ( > G Pa) or soft (0.2 kPa) matrices. Collectively, these results suggest that matrix stiffness governs the expression of β1 integrin and Cav1, which reciprocally control each other, and subsequently determine FA assembly and turnover.

## Introduction

Matrix stiffness exerts substantial effects on various cellular functions, including survival, proliferation, differentiation and migration^1–4^. Soft matrix is considered an inhibitor of proliferation and a promoter of differentiation in renal tubular cells^5^. Paszek *et al*.^6^ showed that increase extracellular matrix (ECM) stiffness disrupted tissue morphogenesis of mammary gland epithelial cells, whereas a decrease in cell tension alleviated the malignant behavior of breast cancer cells^6^. Additionally, matrix crosslink-enhanced ECM tension promotes tumor progression and liver fibrosis^7^. These data suggest a critical role of ECM stiffness in physiology and pathophysiology.

Integrins and the downstream focal adhesion (FA) complex proteins are known as mechanosensors and mechanotransducers that sense and transduce mechanical signals into biochemical signals. In tissues such as mammary gland, liver, and kidney, integrins and FA complex proteins are absent or very weakly expressed, whereas numerous cell lines and primary cells that are grown on tissue culture surfaces express high levels of integrins and FA-related proteins^5,8^. It suggests matrix stiffness has a large impact on the expression of integrin and FA complex. It is acknowledged that increase in matrices stiffness promotes the activation and clustering of integrin, and also FA assembly^9–11^. However, how matrix stiffness control β1 integrin protein levels still largely unclear.

Caveolin-1 (Cav1), a structural protein of caveolae/lipid rafts that conducts and coordinates multiple signals at the cell surface^12,13^. For example, it is well accepted that Cav1 is involved in integrin-dependent signaling^11,14,15^ and FA assembly/turnover^16,17^, and acts as a mechanosensor in sensing flow and stretch-induced mechanotransduction^18,19^. Moreover, the function of Cav1 is highlighted in integrin-mediated ECM remodeling of tumor-associated fibroblasts^20^, and in integrin-dependent invasion and metastasis of tumor cells^16,21^. However, the underlying mechanism by which Cav1 regulates mechanosensation and matrix stiffness-dependent integrin activation remains unclear. In this study, we show a reciprocal regulation between Cav1 and β1 integrin that is orchestrated by matrix stiffness, and highlighted their functions in mechanical sensing machinery and delineated their role in generating platforms at the cell surface for the initiation of FA assembly.

## Results

### Soft matrix reduces cell spreading, FA assembly, and β1 integrin expression

**Figure 1:**
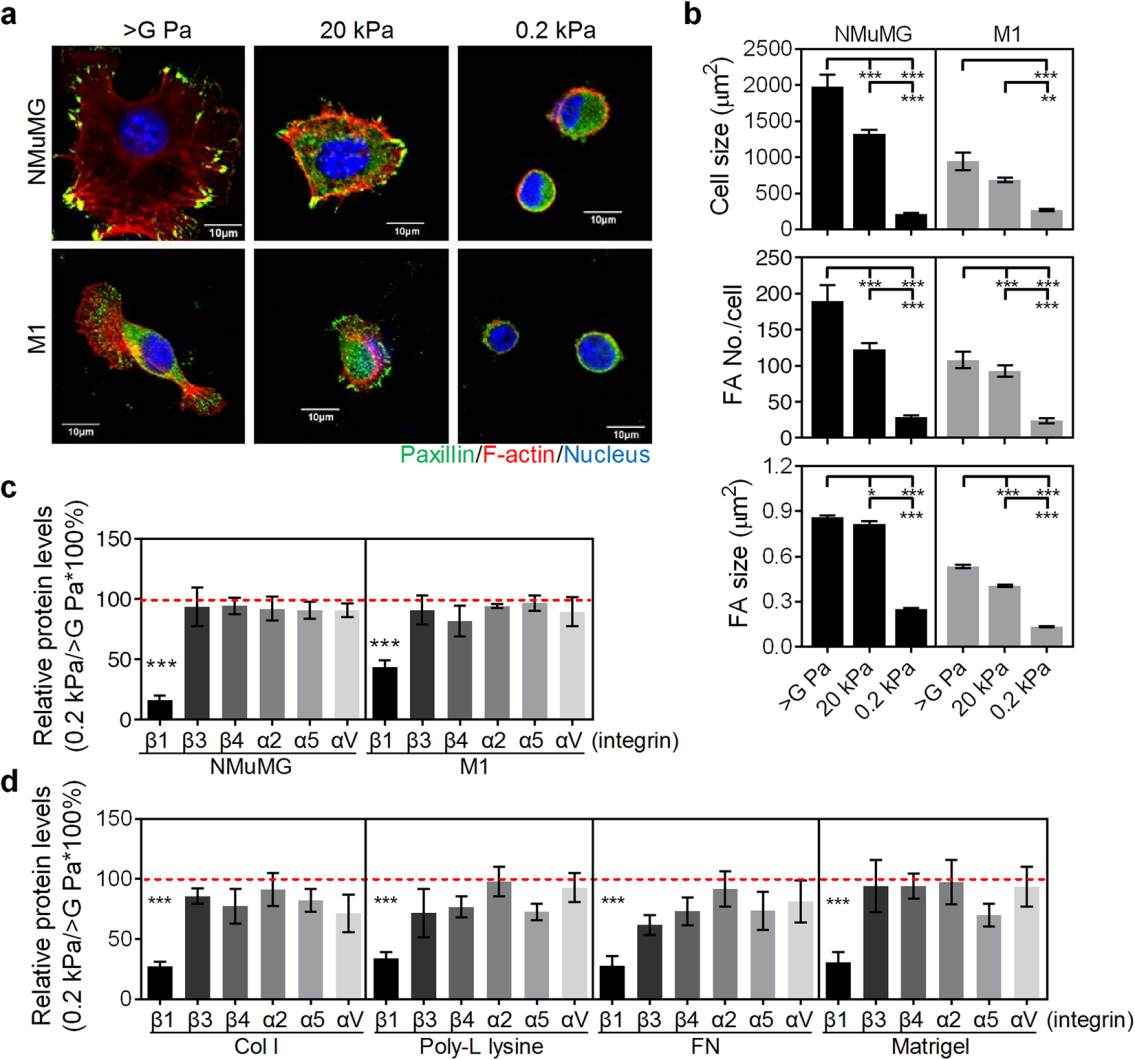
Soft matrix impedes cell spreading and focal adhesion (FA) assembly and selectively suppressed the expression of β1 integrin. NMuMG and M1 cells were grown on type I collagen (Col I)-coated matrices, including culture dishes (E > GPa) as well as PA gels of 20 kPa and 0.2 kPa for 4 h. (**a**) Representative confocal images of cells grown on indicated conditions. Cells were stained for paxillin (green), F-actin (red), and nuclei (blue). Scale bar = 10 μm. (**b**) Quantification results show the average cell size as well as the size and number of FAs in cells grown on indicated matrices. At least 20 representative images from each condition were analyzed. (**c**) Quantification results show the relative protein levels of integrins in cells grown on dishes or 0.2 kPa PA gels. β-actin-normalized data in each condition was compared with those of cells cultured on tissue culture plastic (dotted line). (**d**) Quantification results show the relative protein levels of integrins in NMuMG cells grown on Col I-, poly-L lysine-, fibronectin (FN)-, or Matrigel-coated dishes or 0.2 kPa PA gels. β-actin-normalized data in each condition was compared with those of cells cultured on dishes (dotted line). All data are expressed as relative mean ± SEM from three independent experiments. *p < 0.05; **p < 0.01; ***p < 0.001.

Focal adhesion assembly orchestrates actin cytoskeletal organization, which consequently affects cell spreading, migration, and numerous cellular functions. To correctly determine the effect of matrix stiffness on cell spreading and focal adhesion (FA) assembly, cells were cultured on collagen-coated dishes (>1 GPa) or less stiff polyacrylamide (PA) gels (either 20 or 0.2 kPa). Two epithelial cell lines, NMuMG and M1 cells, which originate from soft tissue were used. Cells grown on collagen-coated dishes displayed the largest cell spreading areas and the greatest sizes and numbers of FAs (Fig. 1a,b). These features declined with decreasing matrix stiffness, from >1 GPa to 0.2 kPa, confirming that matrix stiffness regulates cell spreading and FA assembly. Similar results were also replicated in various epithelial cell lines and fibroblasts (data not shown). Members of the integrin family are known to signal to initiate FA assembly. For this reason, we examined the effect of matrix stiffness on integrins expression and activation. Of the integrins analyzed, we found that β1 integrin protein levels were strongly and specifically downregulated in cells cultured on soft matrix (Fig. 1c), regardless of the substrate coating (collagen, poly-L-lysine, fibronectin, or matrigel) (Fig. 1d).

### A reduction in matrix stiffness leads to decreased β1 integrin protein levels via lysosome degradation

**Figure 2:**
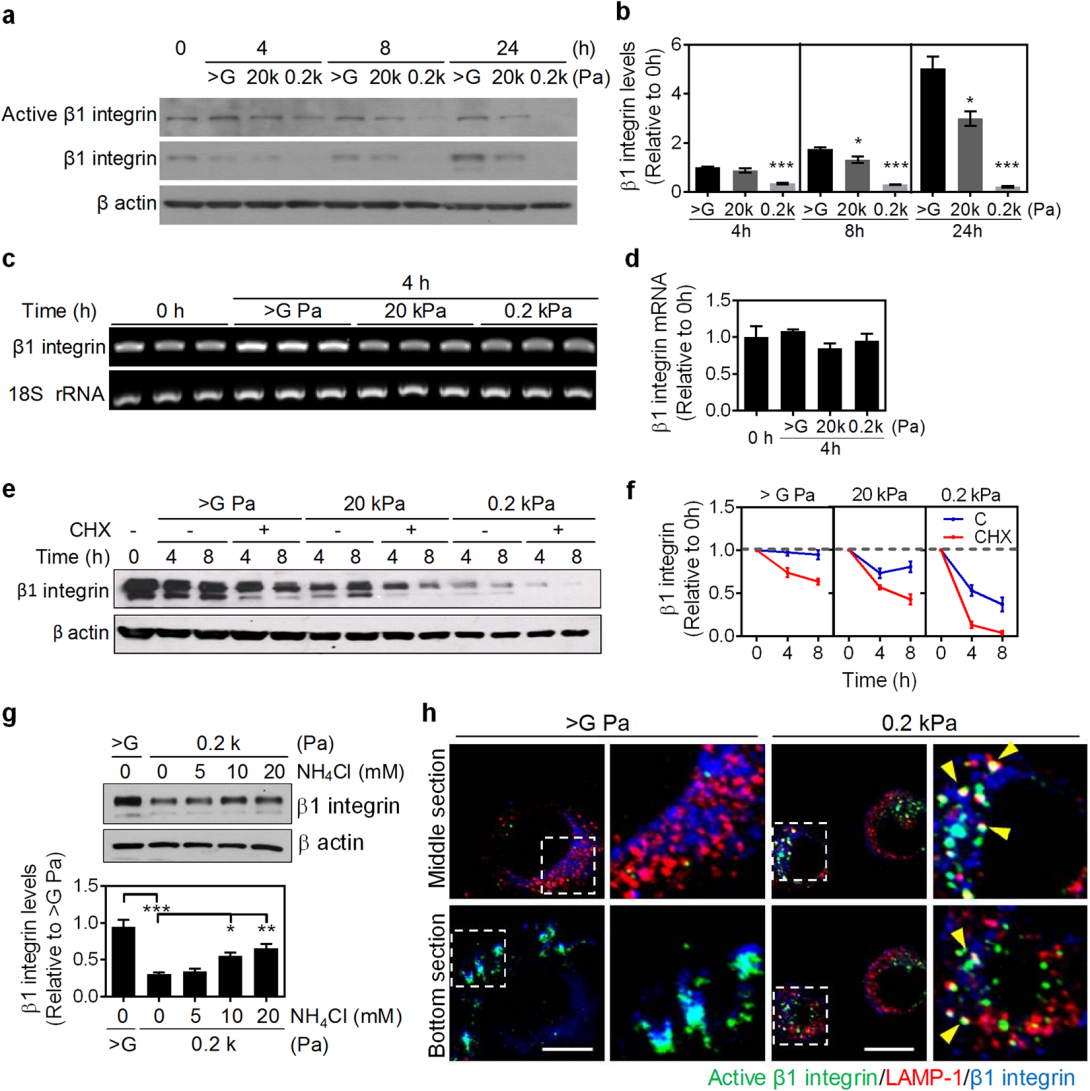
Soft matrix downregulates β1 integrin via lysosome-mediated protein degradation. (**a**) Representative western blot analysis results of NMuMG cells grown on matrices of varying stiffness at the indicated times. The protein levels of active β1 integrin and total β1 integrin were analyzed. β actin was used as an internal control. Also see Supplementary Fig. S3. (**b**) Quantification results of total β1 integrin were from (**a**) and two other experiments. β-actin-normalized data in each condition was compared with those of cells cultured on dishes at 0 h. All data are expressed as relative mean ± SEM from three independent experiments. (**c**) The β1 integrin mRNA levels of NMuMG cells grown on matrices of varying stiffness for 4 h were assessed and the 18 S rRNA is used a loading control. (**d**) Quantification results of β1 integrin mRNA levels from (**c**). (**e**) Representative western blot analysis results of cycloheximide (CHX)-chase assay of β1 integrin turnover in NMuMG cells grown on matrices of varying stiffness. NMuMG cells were pre-treated with or without 50 μg/ml CHX for 30 min before detached from tissue culture plastics. Then, cells were detached (time = 0) with low dose of trypsin and replated on different matrices with or without the sustained CHX treatment for the indicated time. Also see Supplementary Fig. S3. (**f**) Quantitative results of β1 integrin turnover were from (**e**). β-actin-normalized data in each condition was compared with those of cells in suspension at 0 h. (**f**) Top: Representative western blot analysis results of NMuMG cells grown on 0.2 kPa gel and treated with different concentrations of NH_4_Cl. The protein level of integrin β1 was analyzed. β actin was used as an internal control. Bottom: Quantification results of β1 integrin were from three independent experiments. β-actin-normalized data in each condition was compared with those of cells cultured on dishes. Also see Supplementary Fig. S3. (**g**) Representative confocal immunofluorescence images of NMuMG cells grown on the indicated conditions for 4 h. Cells were stained for β1 integrin (blue), active β1 integrin (green), and lysosomal-associated membrane protein-1 (LAMP-1) (red). Scale bar = 10 μm. All data are expressed as relative mean ± SEM from three independent experiments. *p < 0.05; **p < 0.01; ***p < 0.001.

The time-course study showed that the expression of β1 integrin is decreased with reducing matrix stiffness (Fig. 2a,b). This was independent of changes in β1 integrin transcription, as mRNA levels of β1 integrin were constant at all ranges of matrix stiffness (Fig. 2c,d). This suggested that post-transcriptional regulation might contribute to the observed soft matrix elicited reduction of β1 integrin. To analyze the effect of matrix stiffness on protein stability, NMuMG cells were pretreated with or without cycloheximide for 30 minutes to block translation before being detached from culture dishes. The cycloheximide (CHX) pre-treatment for 30 min did not change the protein levels of β1 integrin, which suggests a high protein stability of β1 integrin in cells grown on stiff tissue culture plastics (Fig. S1). Cells were then detached with a low dose of trypsin to reduce the loss of β1 integrin from trypsinization (Fig. S1). After detachment (time = 0), cells were subsequently seeded in the presence of CHX on matrices of varying stiffness for 4 or 8 hours. β1 integrin protein levels remained constant in cells seeded on tissue culture plastic without CHX treatment. Upon CHX treatment, the protein levels of β1 integrin in cells grown on tissue culture plastics were decayed by 26.1% to 36.5% within 4 and 8 hours, respectively. β1 integrin protein levels significantly decreased with declining matrix stiffness and time, which was more evident upon treatment with CHX (Fig. 2e,f). These findings demonstrate that the half-life of β1 integrin protein was decreased in NMuMG cells cultured on soft matrix.

To specify the pathway involved in soft matrix-induced β1 integrin protein turnover, several inhibitors of protein degradation were used. These experiments showed that blocking the lysosomal pathway by NH_4_Cl treatment (which blocks lysosomal acidification, and subsequently degradation) rescued soft matrix-induced β1 integrin downregulation (Fig. 2g). These results indicate that the reduction of β1 integrin in NMuMG cells cultured on soft matrix was mainly through the lysosomal degradation pathway. In NMuMG cells grown on tissue culture plastic (>G Pa), β1 integrin and active β1 integrin, induced by ligand binding and detected by an antibody against the exposed domain upon activation, formed large puncta at basal sites on the cell periphery, with no clearly observed puncta localizing in lysosome-associated membrane protein-1 (LAMP-1) positive compartment (Fig. 2h). In contrast, active β1 integrin and β1 integrin were partially co-localized with LAMP-1 in NMuMG cells cultured on soft matrix, throughout the cell, in different confocal Z-planes. Similar results were obtained with colocalization studies of β1 integrin with early endosome antigen 1 (EEA1) staining (Fig. S2), suggesting soft matrix induced endocytosis of β1 integrin. Although β1 integrin protein levels decreased in NMuMG cells cultured on soft matrix within 4 h, the remaining β1 integrin exhibited highly activation and accumulated in cytosol (Fig. 2a,h). Collectively, these data are consistent with the model whereby protein stability of β1 integrin in cells cultured on soft matrix is decreased due to the rapid internalization of β1 integrin and subsequent lysosome-mediated protein degradation.

### Lipid raft and Cav1 contribute to matrix stiffness-modulated cell spreading, FA assembly, and the activation and expression of β1 integrin

**Figure 3:**
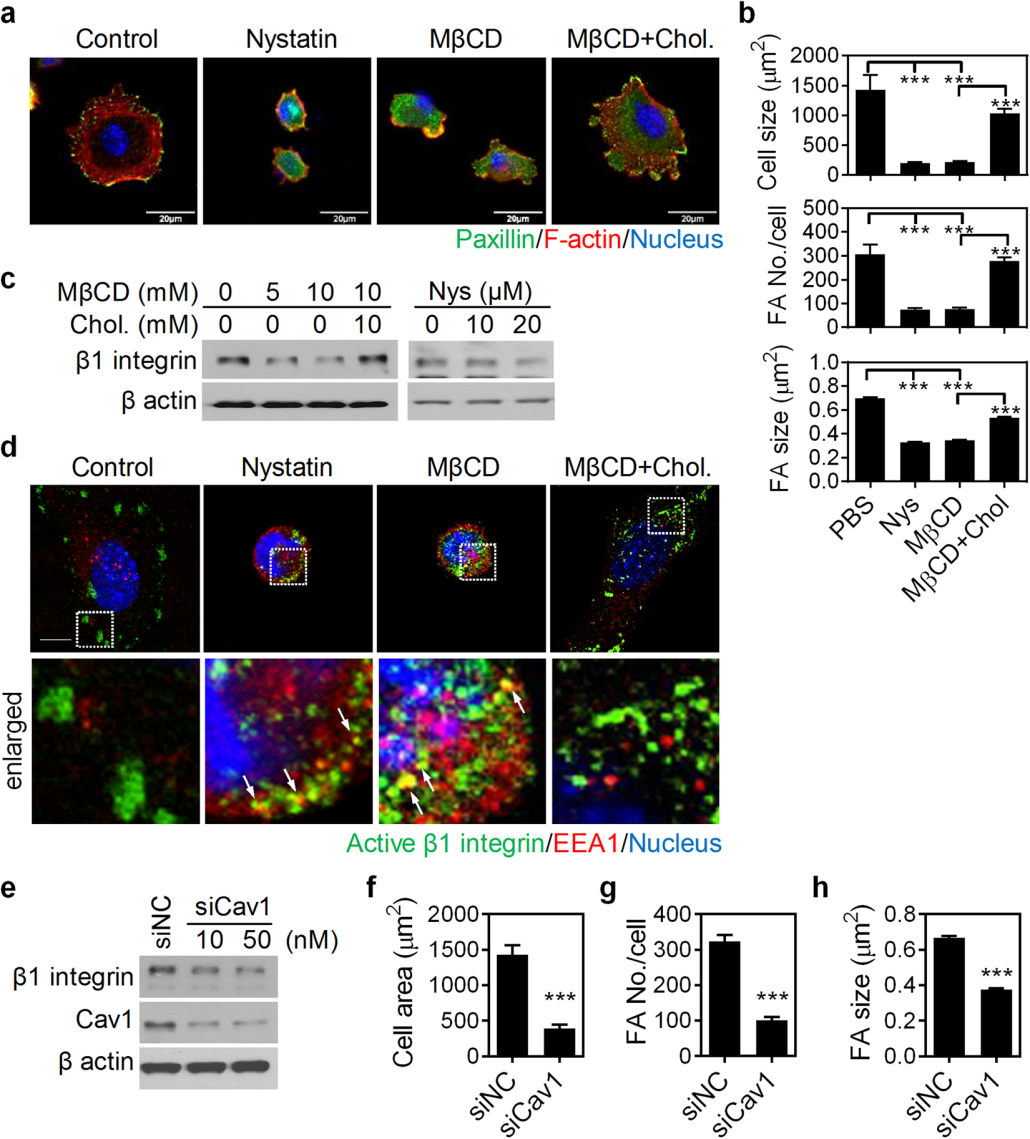
Both disruption of lipid rafts and knockdown of Cav1 impede cell spreading, focal adhesion (FA) assembly, and β1 integrin expression. (**a**) Representative confocal immunofluorescence images of NMuMG cells grown on dishes and treated with 20 μM nystatin, 10 mM methyl-beta-cyclodextrin (MβCD), or 10 mM MβCD plus 10 mM cholesterol (MβCD + Chol.) for 4 h. Cells were stained for paxillin (green), F-actin (red), and nuclei (blue). Scale bar = 20 μm. (**b**) Quantification results show the average cell size as well as the size and number of FAs in cells grown on indicated conditions. At least 10 representative images from each condition were analyzed. (**c**) Representative western blot analysis results of NMuMG cells treated with nystatin, MβCD, or MβCD + Chol. for 4 h. The protein levels of β1 integrin were analyzed. β actin was used as an internal control. Also see Supplementary Fig. S4. (**d**) Representative confocal immunofluorescence images of NMuMG cells grown on the indicated conditions for 4 h. Cells were stained for active β1 integrin (green), early endosome antigen 1 (EEA1) (red), and nuclei (blue). Bar = 10 μm. (**e**) Representative western blot analysis results of NMuMG cells transfected with siNC (negative control) and siCav1. Also see Supplementary Fig. S4. The protein levels of β1 integrin and Cav1 were analyzed. β actin was used as an internal control. (f) Quantification results show the average cell size as well as the size and numbers of FA in cells transfected with siNC and siCav1. At least 10 representative images from each condition were analyzed. All data are expressed as relative mean ± SEM. *p < 0.05; **p < 0.01; ***p < 0.001.

**Figure 4:**
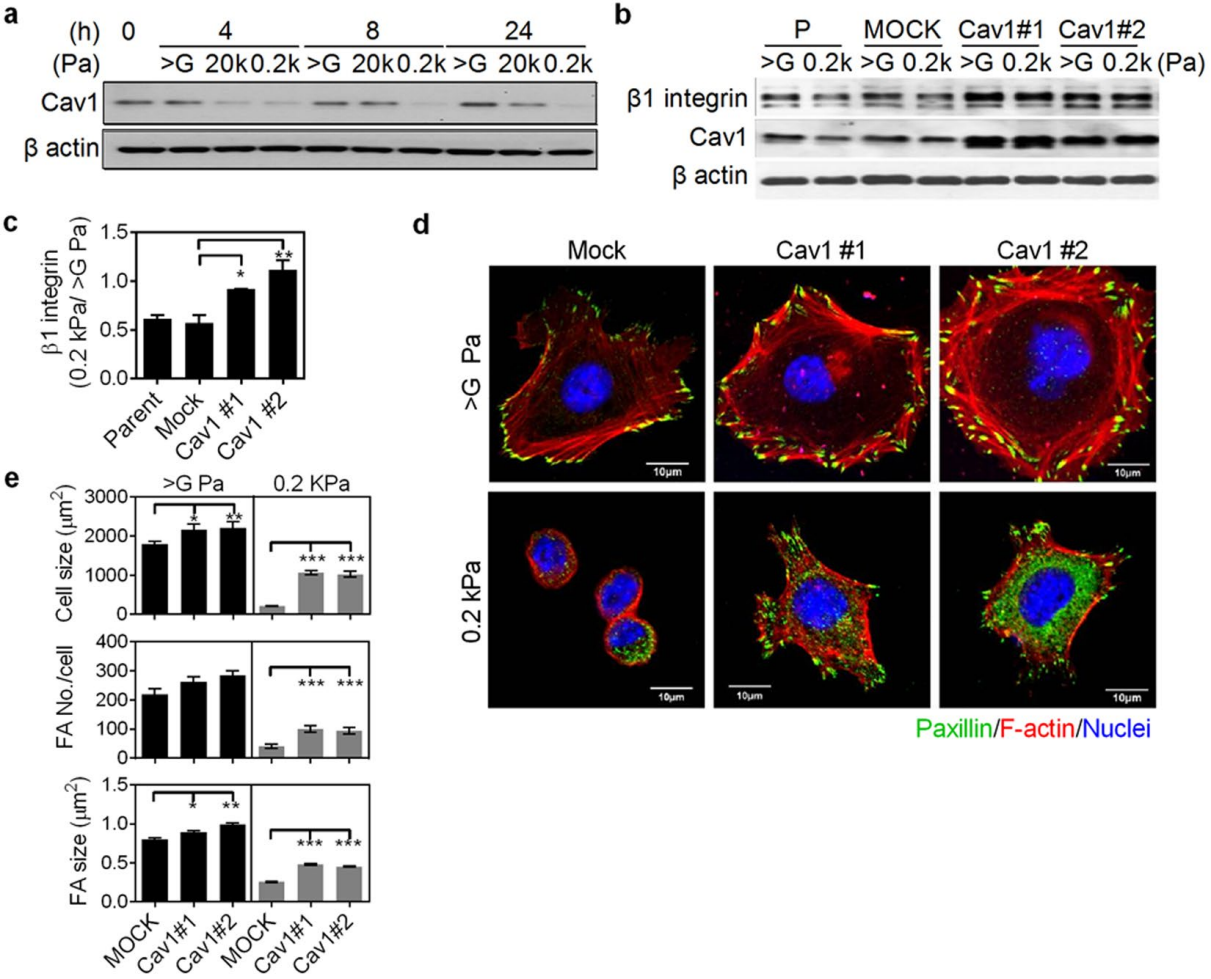
Caveolin-1 (Cav1) regulates cell spreading and focal adhesion (FA) assembly via modulation of protein levels of β1 integrin. (**a**) Representative western blot analysis results of NMuMG cells grown on matrices of varying stiffness for the indicated times. The protein levels of Cav1 were analyzed. Also see Supplementary Fig. S5. (**b**) Representative western blot analysis results of control vector (mock) - or Cav1-transfected NMuMG cells grown on matrices of varying stiffness for 4 h. The protein levels of β1 integrin and Cav1 were analyzed. Also see Supplementary Fig. S5. (**c**) Quantitative results of β1 integrin were from (**b**) and two other experiments. β-actin-normalized data in each condition was compared with those of cells grown on tissue culture plastic (>G Pa). (**d**) Representative confocal immunofluorescence images of mock- or Cav1-transfected NMuMG cells grown under indicated conditions for 4 h. Cells were stained for paxillin (green), F-actin (red), and nuclei (blue). (**e**) Quantification results show the average cell size as well as the size and number of FAs in cells grown on indicated conditions. At least 10 representative images from each condition were analyzed. All data are expressed as relative mean ± SEM. *p < 0.05; **p < 0.01; ***p < 0.001.

Cav1 and lipid rafts are critical in β1 integrin activation/clustering^22^, FA assembly and turnover^23–25^, cell adhesion, migration and survival^17,20,26^. Therefore, we tested the role of Cav1 and lipid rafts in matrix stiffness-modulated FA assembly and β1 integrin protein turnover. Disruption of lipid rafts with MβCD or nystatin decreased cell spreading, FA assembly, and β1 integrin protein levels in NMuMG cells cultured on dishes (Fig. 3a-c). Disruption of lipid rafts also facilitated the internalization of active β1 integrin, which partially co-localized with the early endosomal marker, EEA1 (Fig. 3d). As expected, supplementation of the medium with cholesterol blocked the observed effect of MβCD on β1 integrin downregulation (Fig. 3a,c,d). Knockdown of Cav1 by siRNA in NMuMG cells cultured on tissue culture plastic resulted in a reduction of β1 integrin protein levels, cell spreading, and FA assembly (Fig. 3e,f). Together, both lipid raft and Cav1 are critical in regulating cell spreading, FA assembly, and the protein levels of β1 integrin; more specifically, it implies that cholesterol-rich domains and Cav1 stabilize β1 integrin levels at the cell surface. Supporting this, first, Cav1 protein levels significantly decreased with declining matrix stiffness in NMuMG cells during culture (Fig. 4a). Second, overexpression of Cav1 not only increased β1 integrin protein, cell spreading, and FA assembly in NMuMG cells cultured on dish (>G Pa), but also averted soft matrix-decreased β1 integrin protein as well as cell spreading and FA assembly (Fig. 4b-e). These results indicate that the level of Cav1 is critical in the regulation of β1 integrin turnover.

### Phosphocaveolin-1 positively controls cell spreading, FA assembly, and β1 integrin protein levels

**Figure 5:**
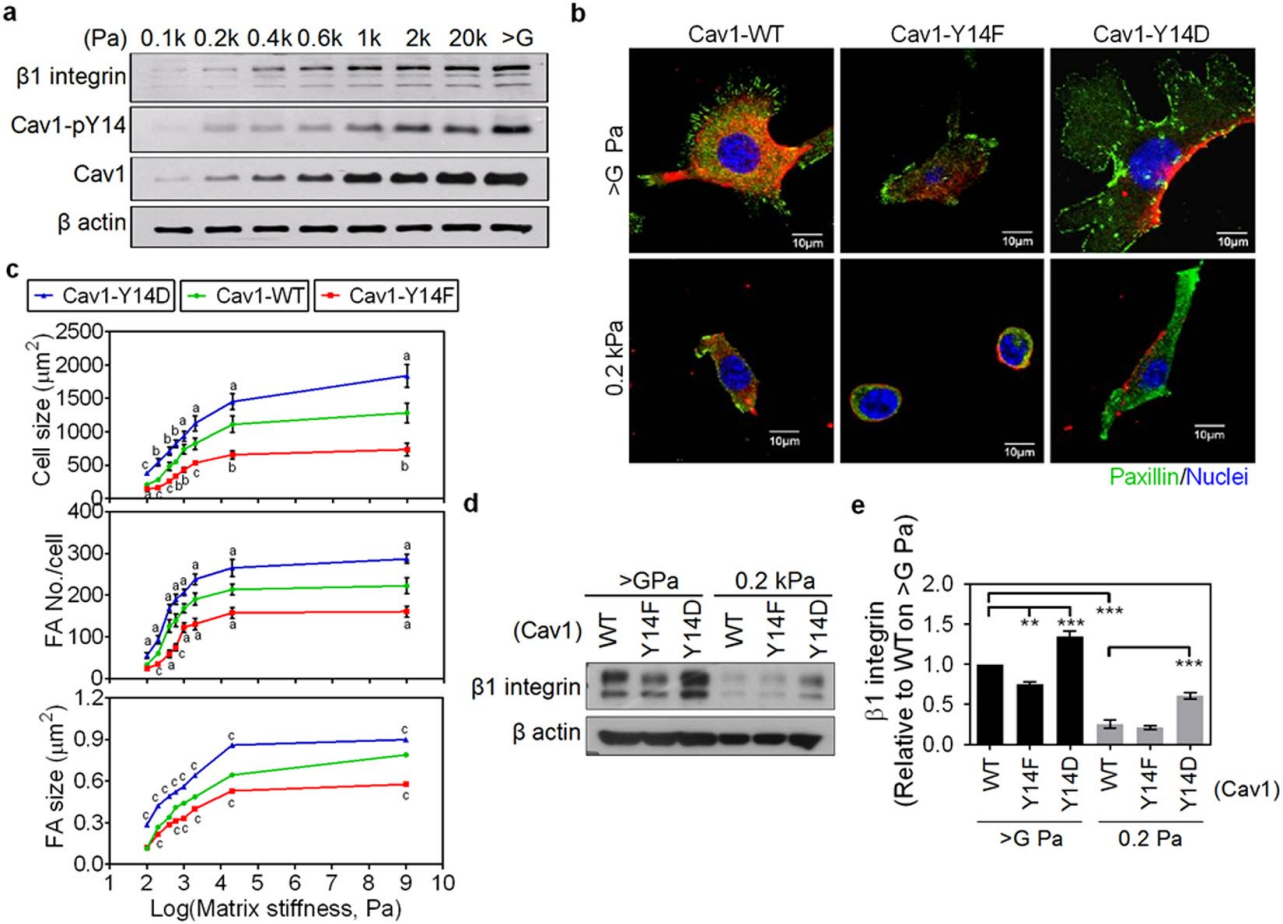
Phosphorylation of caveolin-1 is required for β1 integrin protein stabilization, focal adhesion (FA) assembly and cell spreading. (**a**) Representative western blot analysis results of NMuMG cells grown on matrices of varying stiffness for 4 h. The protein levels of integrin β1, Cav1, and Cav1-pY14 were analyzed. Also see Supplementary Fig. S6. (**b**) Representative confocal images of Cav1-WT-RFP-, Cav1-Y14F-RFP-, or Cav1-Y14D-RFP-transfected NMuMG cells grown on matrices of varying stiffness for 4 h. Cells were stained for paxillin (green) and nuclei (blue). Scale bar = 10 μm. (**c**) Quantification results show the average cell size as well as the size and number of FAs in Cav1-WT-RFP-, Cav1-Y14F-RFP-, or Cav1-Y14D-RFP-transfected NMuMG cells grown on matrices of varying stiffness for 4 h. At least 10 representative images for each condition were used for analysis. (**d**) Representative western blot analysis results of Cav1-WT-RFP-, Cav1-Y14F-RFP-, or Cav1-Y14D-RFP-transfected NMuMG cells grown on indicated conditions for 4 h. The protein levels of integrin β1 were analyzed. Also see Supplementary Fig. S6. (**e**) Quantitative results of β1 integrin were from (**d**) and two other experiments. β-actin-normalized data in each condition was compared with those of Cav1-WT-RFP-transfected NMuMG cells grown on dishes (>G Pa). Each bar represents mean ± SEM. All data are expressed as relative mean ± SEM. a: *p < 0.05; b: **p < 0.01; c: ***p < 0.001.

Phosphorylation of Cav1 on tyrosine 14 (pY14-Cav1) has been reported to regulate its mechanotransduction properties, with a functional effect on actin cytoskeleton and Src-induced FA assembly^16,21,27^. NMuMG cells grown on matrices of increasing stiffness showed a proportional increase not only in endogenous Cav1 and β1 integrin, but also in endogenous pY14-Cav1 (Fig. 5a). The representative images showed that overexpression of phospho-deficient mutant Cav1 (Cav1-Y14F) impeded cell spreading and FA assembly, even in NMuMG cells grown on stiff matrix (Fig. 5b, middle). In contrast, overexpression of a phosphomimicking mutant Cav1 (Cav1-Y14D) significantly increased cell spreading area and FA assembly in NMuMG cells cultured on both stiff and soft matrix (Fig. 5b, right). The cell spreading areas, and the sizes and numbers of FAs formed sigmoid curves with the increasing matrix stiffness (Fig. 5c). Notably, the sigmoidal curves were shifted downward or upward by Cav1-Y14F or by Cav1-Y14D, respectively (Fig. 5c). These data indicate a positive effect of phospho-Cav1 on matrix stiffness-regulated cell spreading and FA assembly. We then test whether the phosphorylation of Cav1 affects matrix stiffness-regulated β1 integrin protein turnover. Cav1-Y14F overexpression significantly reduced, whereas Cav1-Y14D overexpression significantly increased the protein levels of β1 integrin in NMuMG cells cultured on stiff matrix (Fig. 5d). As expected, the soft matrix-induced decrease in β1 integrin was partially rescued in NMuMG cells transfected with Cav1-Y14D (Fig. 5d). Taken together, Cav1-Y14 phosphorylation is mechanistically important in Cav1-regulated protein turnover of β1 integrin, cell spreading and FA assembly.

### β1 integrin-mediated adhesion signal is required for the expression of Cav1 and membrane recycling of lipid rafts

**Figure 6:**
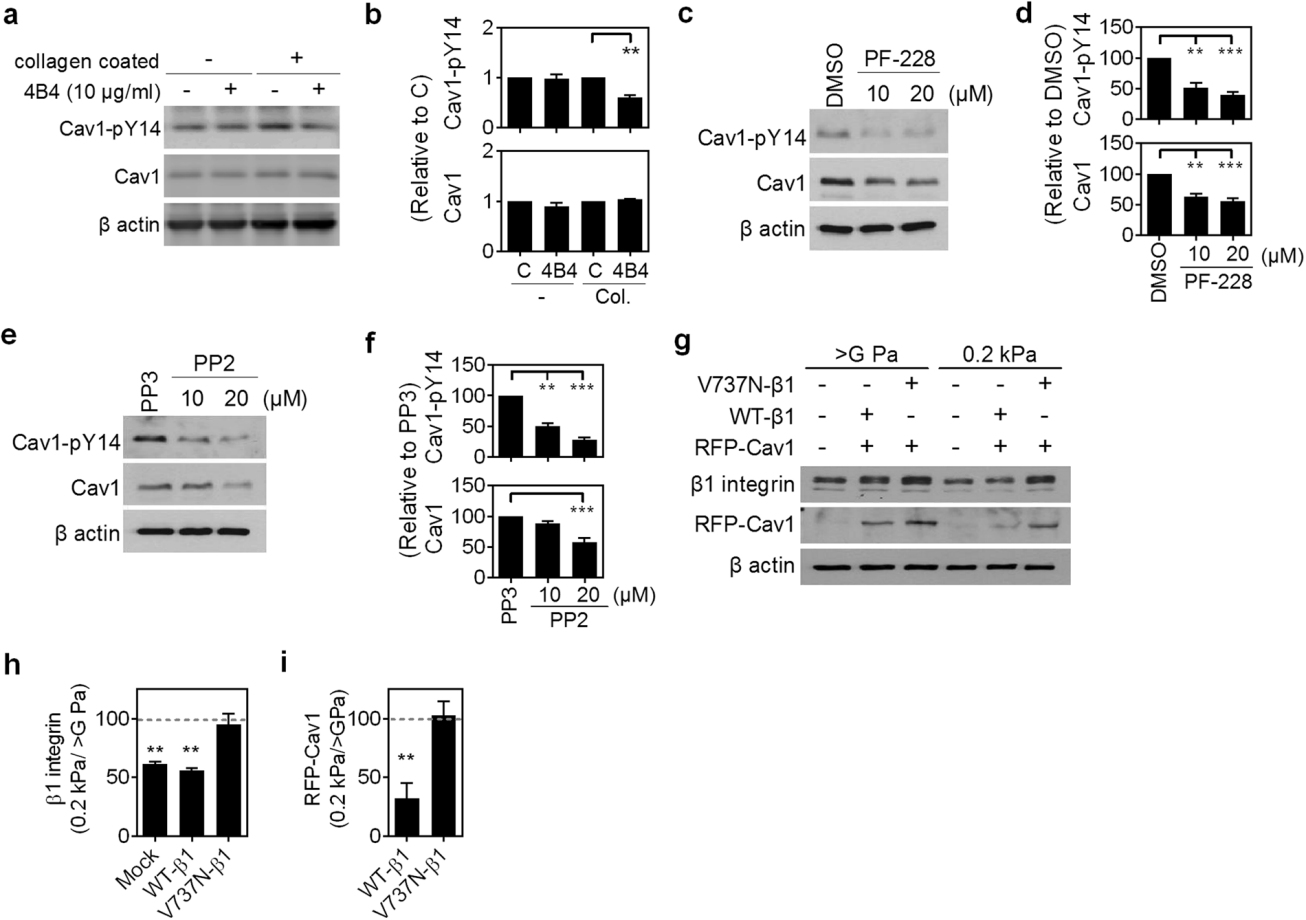
Integrin β1-mediated adhesion signal through FAK/Src activity controls the protein and phosphorylation levels of caveolin-1. (**a**) NMuMG cells were pretreated with or without the β1 integrin blocking antibody, 4B4, for 30 min. Cell were then replated on tissue culture plastics with or without collagen coating with the continuous treatment of blocking antibody for another 4 h. The protein and phosphorylation levels of Cav1 were assessed by western blot. Also see Supplementary Fig. S7. (**b**) Quantitation of results of Cav1 and Cav1-pY14 were from (**a**) and two other experiments. β-actin-normalized data in each condition was compared with those of cells without 4B4 treatment (C). (**c**) Representative western blot analysis results of NMuMG cells grown on tissue culture plastic treated with DMSO or indicated concentrations of PF-228 (μM) for 4 h. The protein and phosphorylation levels of caveolin-1 were analyzed. Also see Supplementary Fig. S7. (**d**) Quantitation of results of Cav1 and Cav1-pY14 were from (**c**) and two other experiments. β-actin-normalized data in each condition was compared with those of cells treated DMSO. (**e**) Representative western blot analysis results of NMuMG cells grown on tissue culture plastic treated with PP3 or indicated concentrations of PP2 (μM) for 4 h. The protein and phosphorylation levels of caveolin-1 were analyzed. Also see Supplementary Fig. S7. (**f**) Quantitative results of Cav1 and Cav1-pY14 were from (**e**) and two other experiments. β-actin-normalized data in each condition was compared with those of cells treated PP3. (**g**) Representative western blot analysis results of HEK293T cells grown on dishes (>G Pa) or 0.2 kPa PA gel for 4 h. HEK293T cells were cotransfected with wild-type β1 integrin (WT-β1) or auto-clustering β1 integrin (V737N-β1) and with or without wild-type Cav1 (Cav1). The protein levels of β1 integrin and Cav1 were analyzed. Also see Supplementary Fig. S7. Quantitative results of β1 integrin (**h**), or exogenous Cav1 (i) were from (**g**) and two other experiments. β-actin-normalized data in each condition was compared with those of cells grown on dishes (>G Pa). All data are expressed as relative mean ± SEM from three independent experiments. *p < 0.05; **p < 0.01; ***p < 0.001.

Considering the critical role of Cav1-Y14 phosphorylation in mechanical sensing as well as focal adhesion assembly and β1 integrin protein turnover, we next investigated the upstream signals driving Y14 phosphorylation of Cav1. It has been reported that adhesion signals through FAK or Src activity lead to Cav1 phosphorylation^21^. To test whether this holds true in our model system, NMuMG cells were pretreated with or without β1 integrin blocking antibody, 4B4 for 30 min, and then replated on tissue culture plastics coated with or without collagen. The 4B4 treatment reduced the collagen-induced Y14 phosphorylation of Cav1, suggests β1 integrin-induced adhesion signal is the upstream signal driving pY14-Cav1 (Fig. 6a,b). NMuMG cells treated with the FAK or Src inhibitors, PF-573228 or PP2, respectively, showed a reduction in both pY14-Cav1 and Cav1 (Fig. 6c-f). These data indicate that β1 integrin-mediated adhesion signals, through FAK and Src activity, control the phosphorylation and expression of Cav1.

Previous study showed that the increase in β1 integrin clustering increases FAK phosphorylation in cells grown on soft matrix or within a tissue context^11,28^. To examine whether soft matrix reduced Cav1 protein levels occur via diminishing β1 integrin-dependent adhesion signaling, HEK293T cells were either cotransfected with Cav1-WT and wild-type β1 (WT-β1) integrin, or Cav1-WT and auto-clustering β1 (V737N-β1) integrin. The protein levels of total β1 integrin were decreased in cells grown on soft matrix; however, this phenomenon was reduced by V737N-β1 overexpression (Fig. 6g,h). The reduction of exogenous Cav1 found in cells cultured on soft matrix while co-expressed WT-β1 integrin (Fig. 6i) was also reduced by co-expression of with V737N-β1 integrin (Fig. 6h). The inhibition of downregulation of endogenous Cav1 on soft matrix by V737N-β1 integrin, but not WT-β1 integrin, was further confirmed with cells without expression of exogenous Cav1 (data not shown). These results indicate that β1 integrin-dependent adhesion signaling plays a decisive role in matrix stiffness-modulated Cav1 protein turnover, and suggest that reciprocal regulation between β1 integrin and Cav1 exists.

### Reciprocal regulation of β1 integrin and Cav1 by matrix stiffness modulates cell spreading and FA assembly

**Figure 7:**
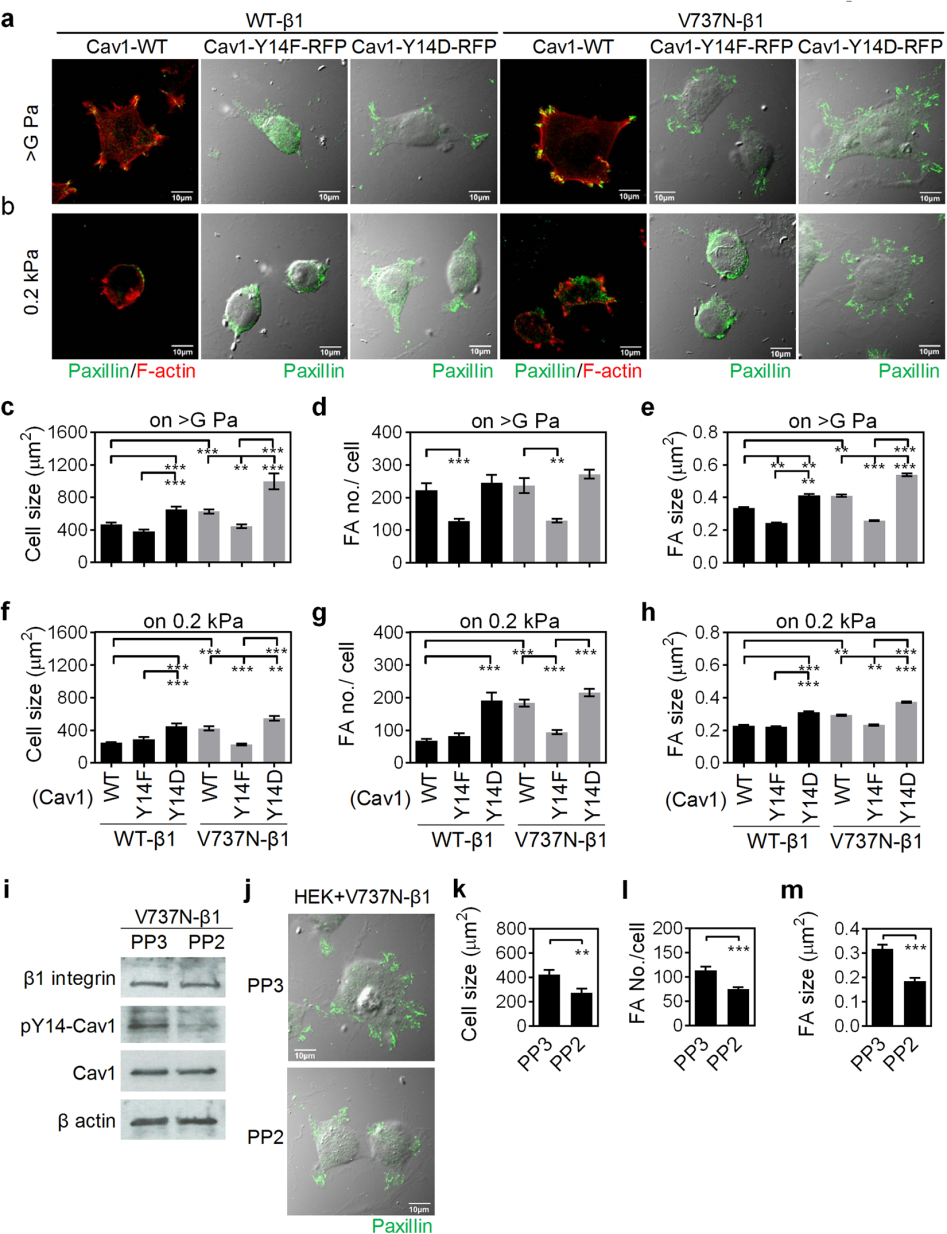
The synergistic effect of β1 integrin and caveolin-1 (Cav1) in cell spreading and focal adhesion (FA) assembly. HEK293T cells were cotransfected with wild-type β1 integrin (WT-β1) or auto-clustering β1 integrin (V737N-β1), and Cav1-WT (WT), Cav1-Y14F (Y14F), or Cav1-Y14D (Y14D). (**a** and **b**) Representative confocal immunofluorescence images of HEK293T cells transfected and grown in indicated conditions. Cells were stained for paxillin (green) and F-actin (red). Scale bar = 10 μm. (**c**–**h**) Quantification results show the average cell size (**c** and **f**) as well as the numbers (**d** and **g**) and size (**e** and **h**) of FAs in HEK293T cells transfected and grown in indicated conditions. All data are expressed as relative mean ± SEM. *p < 0.05; **p < 0.01; ***p < 0.001. (**i**) HEK293T cells co-transfected with V737N β1 integrin and Cav1-WT for 24 h were treated with 10 μM PP3 or PP2. The protein levels of β1 integrin, pY14-Cav1, and Cav1 were assessed by western blot. Also see Supplementary Fig. S8. (**j**) Representative images showed the effects of Src inhibition on the cell phenotypic change. HEK293T cells were co-transfected with V737N β1 integrin and Cav1-WT. (**k**–**m**) The quantification results showed the cell size (**k**), FA no./cell (**l**) and FA size (**m**), of HEK293T cells transfected with V737N β1 integrin and Cav1-WT and then treated with PP3 or PP2.

To test this reciprocal regulation between β1 integrin and Cav1 in cell spreading and FA assembly, HEK293T cells were co-transfected with the combinations of β1 integrin mutants (WT-β1 and V737N-β) and Cav1 mutants (Cav1-WT, Cav1-Y14F, or Cav1-Y14D). In cells grown on tissue culture plastic, spreading area and FA assembly were decreased in cells co-transfected with WT-β1 and Cav1-Y14F, and were increased in cells co-transfected with WT-β1 and Cav1-Y14D or V737N-β1 and Cav1-WT, compared to those transfected with WT-β1 and Cav1-WT (Fig. 7a,c-e). Soft matrix decreased cell spreading and FA assembly were rescued in cells co-transfected with WT-β1 and Cav1-Y14D or V737N-β1 and Cav1-WT (Fig. 7b,f-h). Regardless of matrix stiffness, the increases in spreading area and FA assembly caused by co-transfection of V737N-β1 and Cav1-WT were abolished or greatly promoted when Cav1-Y14F or Cav1-Y14D, respectively, were used instead of Cav1-WT (Fig. 7a-h). To test if the V737N-β1-induced cell spreading and Cav1 phosphorylation is Src dependent, cells co-transfected with V737N-β1 and Cav1-WT were treated with PP3 or PP2. The pY14-Cav1 signal was largely reduced upon PP2 treatment (Fig. 7i). The inhibition of Src activity also inhibited V737N-β1-induced cell spreading and FA assembly (Fig. 7j-m). In summary, these data show that β1 integrin clustering and Cav1-Y14 phosphorylation are regulated by matrix stiffness, and exert reciprocal influences on one other, combining to provide synergistic effects on cell spreading and FA assembly.

## Discussion

**Figure 8:**
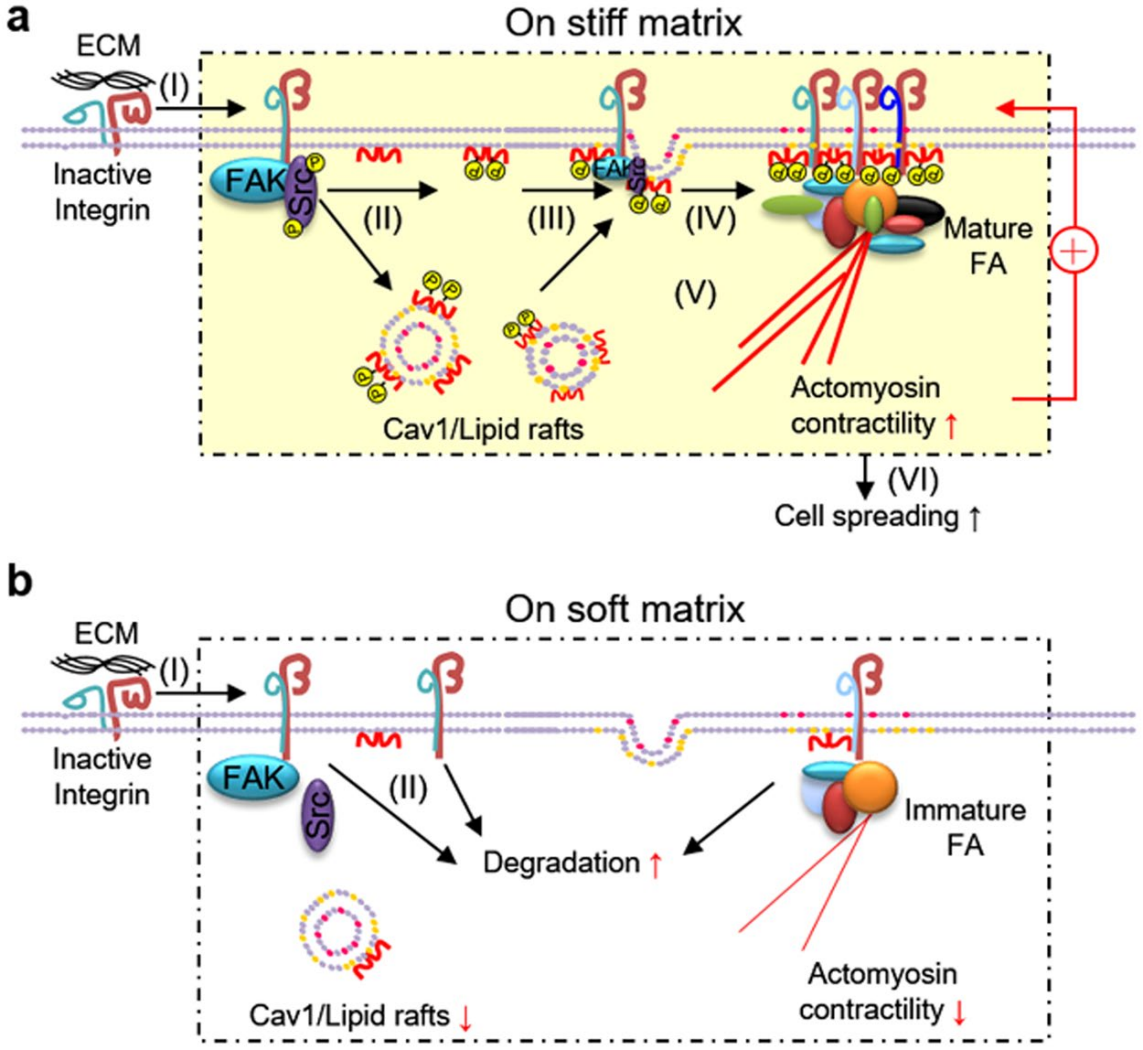
Schematic diagram illustrates the reciprocal regulation between β1 integrin and Cav1 at varying matrix stiffness. (**a**) Extracellular matrix binding to the extracellular portion of the integrin receptor causes its conformation change and the activation of FAK/Src in cells grown on stiff matrix (I). Activated Src and FAK subsequently increases the phosphorylation of Cav1 (II), which triggers the membrane expression/stability of lipid rafts (III). Lipid rafts in turn provide inside-out signals to support the stability and clustering of β1 integrin, which subsequently promotes focal adhesion (FA) assembly and actin organization (IV). Actomyosin-generated force provides a positive feedback loop to reinforce the clustering of β1 integrin and FA assembly. Meanwhile, β1 integrin clustering reciprocally facilitates the membrane expression of Cav1/lipid rafts (V). Finally, cells spread out on stiff matrix (VI). (**b**) Although extracellular matrix binding to the extracellular portion of the integrin receptor causes a conformational change (I), the propagation of intracellular signals is impeded in cells grown on soft matrix. In addition, both β1 integrin and Cav1 are downregulated. Lack of the reinforcement of β1 integrin clustering and FA assembly by actomyosin contractility eventually leads to cells that fail to spread on soft matrix.

In this study, we unravel a possible mechanism that controls β1 integrin activation and clustering during cell adhesion. The persistent activation of β1 integrin is a matrix stiffness-dependent process, which triggers the activation of FAK and Src. These integrin-mediated adhesion signals then trigger the phosphorylation of Cav1 on tyrosine 14, which evokes and stabilizes a biophysical sensing machinery that is associated with lipid rafts membrane recycling. The recruitment of lipid rafts provides inside-out signals to support the stability and clustering of β1 integrin, which subsequently promotes FA assembly and actin organization. Actomyosin-generated forces exerted on actin cytoskeleton provide a positive feedback loop to reinforce the clustering of β1 integrin and FA assembly^11,29,30^. Meanwhile, β1 integrin clustering reciprocally facilitates and maintains the membrane expression of Cav1/lipid rafts. As a result, cells spread out on stiff matrix (Fig. 8a), but this is restricted on soft matrix (Fig. 8b). In summary, the reciprocal regulation of β1 integrin and Cav1/lipid rafts is matrix-stiffness-dependent and is critical to FA assembly and cell spreading.

The effect of V737N mutant in increasing cell extension through expanding adhesion signal has been well demonstrated^6,11,28^. The increase in pY397-FAK was demonstrated in V737N β1 integrin transfected cells grown on soft matrix^4^, and in the V737N transgenic mice^28^. Although V737N increases the adhesion signal on soft matrix, the cell size, or the size and numbers of focal adhesion are still significantly different from those in cells grown on tissue culture plastic. This indicates that even increase in the mechanical initiation sensor, i.e. integrin activation/clustering, is not able to induce the maximum tension or the maturation of focal adhesion if the external environment cannot support the tension. This can be explained by the idea that focal adhesion assembly is the result of a series of mechanical cycles and the traction force exerted on the focal adhesion is the driving force in focal adhesion maturation. Numerous proteins and checkpoints are involved in this regulation^31,32^. As V737N β1 integrin induced FA assembly is matrix density dependent (Fig. 6), it is very likely that V737N β1 integrin-induced pY14-Cav1 is also in a matrix density dependent manner. It also suggests that V737N mutant will only expand the mechanical signal in a certain range under a particular rigidity, given that it is only part of the mechanosensor.

Activity and protein stability of integrins are tightly regulated through their binding to signaling molecules inside the cell. Among these cytosolic molecules, binding with talins and kindlins to the cytosolic domain of integrins is known to be a key step in triggering integrin activation and has a large effect on the physiological functions of integrins^33,34^. Talin and kindlin are involved in the activation of αIIβ3 integrin, and β1 and β2 integrin in circulating platelets or lymphocytes, which has been shown to be crucial for platelet aggregation and thrombus formation of platelets, as well as cell adhesion and infiltration of lymphocytes^35–37^. Fibroblasts lacking either talin or kindlin fail to activate β1 integrin, adhere to fibronectin or maintain Mn^2+^-induced high-affinity integrin conformation^38^. Disruption of the talin binding site in the cytosolic domain of β1 integrin impeded β1 integrin activation, as well as α5β1 integrin-mediated cell scattering, migration and fibronectin fibrillogenesis. Disruption of the kindlin binding site in the cytosolic domain of β1 integrin promoted degradation of α5β1 in late endosomes/lysosomes^39^. In cells grown on soft substrate, we found that both activation and expression of integrin β1 were significantly reduced (Fig. 2). Considering the importance of talin and kindlin in integrin activation and expression, it will be interesting to investigate whether these two proteins could be modulated by matrix stiffness. It was reported that lipid rafts have mechanosensitive properties and raft mobility contributes to the earliest events related to integrin activation at FAs^40,41^. Whether matrix stiffness-modulated Cav-1/lipid rafts contribute to the interactions of talin or kindlin with β1 integrin needs to be further studied. In addition to positive regulators, negative regulators are reported to control integrin activation. These negative regulators might function as clasp stabilizers to keep integrin in its inactive form, compete for the binding site of talin or kindlin in the cytosolic domain of integrin β subunits, or alter integrin trafficking to downregulate the amount of β1 integrin at the plasma membrane^42^. It is possible that matrix stiffness regulates the activation and expression of β1 integrin by switching between positive regulators and negative regulators. Whether negative regulators are involved in soft matrix-downregulated β1 integrin activation and expression needs to be further investigated.

Changes in physical properties of ECMs alter cellular characteristics and threaten tissue functionality^43–45^. Integrin-mediated mechanotransduction plays an important role in numerous biological processes, such as development, maintenance of tissue homeostasis and immunological responses. Aberrant integrin activation or expression is associated with many diseases, such as chronic inflammation, immune deficiencies, and cancer^46,47^. Increases in β1 integrin have been demonstrated in malignant breast cancer cells^48^, and is a prognostic factor for poor overall survival rate of non-small cell lung carcinoma^49,50^. Conversely, an increase in β1 integrin levels is associated with the progression of fibrotic disease, in tissues such as the kidney and liver, and inhibition of β1 integrin signaling or its downstream signals attenuate fibrosis^8,51,52^. It seems plausible that tissue stiffening, due to increased extracellular matrix deposition or crosslinking triggered by TGFβ signaling in fibrotic diseases, triggers β1 integrin signaling, and vice versa. This negative cycle ultimately leads to clinical deterioration. Understanding how stiffening matrix augments β1 integrin expression might therefore provide insights for developing therapeutic strategies for fibrotic diseases. In this study, we highlight the critical roles of Cav1 in β1 integrin-mediated mechanotransduction. We predict that Cav1-induced lipid rafts expression could be the rate-limiting step, not only because its expression and phosphorylation control the protein stability of β1 integrin but also because its membrane expression provides a specialised membrane domain for the organization of β1 integrin-mediated focal adhesions. Suppression of Cav1/lipid rafts expression and activation could turn out to be a crucial antifibrotic strategy.

## Materials and Methods

### Cell cultures and treatment

Mouse mammary gland epithelial cells (NMuMG), collecting duct tubular epithelial cells (M1), and HEK293T were maintained in low-glucose Dulbecco’s modified Eagle’s medium (DMEM, Sigma-Aldrich, St. Louis, MO) supplemented with 10% fetal bovine serum (Invitrogen), 100 IU/ml penicillin (Sigma-Aldrich) and 100 μg/ml streptomycin (Sigma-Aldrich). In experiments with inhibitor treatments, NMuMG cells were pre-treated with inhibitor for 30 min. Then, cells were replated and treated with inhibitors for the indicated times. The inhibitors included: cycloheximide (CHX), nystatin, NH_4_Cl, methyl-beta-cyclodextrin (MβCD), cholesterol (Sigma-Aldrich) and PP2, PP3 (Merck Biosciences). To preserve the membrane-bound β1 integrin and reduce the variances from trypsin-induced β1 integrin cleavage, a low dose of trypsin (0.01%) but high dose of EDTA (1 mM) were used to detach cells from tissue culture plastics.

### Preparation of polyacrylamide (PA) gel with varying stiffness

PA gels of varying stiffness were prepared according to Chen *et al*.^53,54^. Briefly, hydrogels were cast between a SIGMACOTE® (Sigma-Aldrich)-activated glass coverslip and a 3-amino-propyl-trimethoxysilane (Sigma-Aldrich)-activated glass coverslip. To create PA gels of different stiffness, varying concentrations of acrylamide (Sigma-Aldrich) and bis-acrylamide (Sigma-Aldrich) were mixed with acrylic acid (final 0.3%), 10% ammonium persulfate, and TEMED. After polymerization, the PA-gel surface was activated by EDC [1-ethyl-3-(3-dimethylaminopropyl) carbodiimide hydrochloride] (Pierce Biotech, Rockford, Illinois). After extensive washing with 0.1 M MES [2-(N-morpholino) ethanesulfonic acid], 100 μg/ml type I collagen (BD Biosciences PharMingen) in 0.1 M MES was applied to the PA gel and incubated at 4 °C overnight. Finally, PA gels were rinsed well with PBS and soaked in culture medium before use. The mechanical properties of PA gels for each polymerization batch were checked by AFM.

### RNA isolation and reverse-transcription polymerase chain reaction (RT-PCR)

Total RNA was isolated with the Trizol reagent (Invitrogen) according to the manufacturer’s instructions. The extracted RNA were treated with RNase free-DNase I (Invitrogen) to exclude DNA contamination and then reverse transcribed by Moloney murine leukemia virus (MMLV) (Promega, Madison, WI, USA). PCR was performed with specific primer sets at 94 °C for 5 min, followed by 25 cycles at 94 °C for 30 sec, 60 °C for 30 sec, 72 °C for 30 sec, and a final step at 72 °C for 10 min. The PCR products were separated on a 1.2% agarose gel containing ethidium bromide and visualized under a UV transilluminator. The forward and reverse primers for Integrin β1 were 5′-GCCAGGGCTGGTTATACAGA-3′ and 5′-TCACAATGGCACACAGGTTT-3′, respectively. 18 S rRNA was used as internal control. The forward and reverse primers for 18 S rRNA were 5′-TTCCGATAACGAACGAGACTCT-3′ and 5′-TGGCTGAACGCCACTTGTC-3′, respectively.

### Plasmid constructs and transfection

The Cav1 constructs, including wild type (Cav1-WT), phospho-deficient mutant (Cav1-Y14F), and phosphomimicking mutant (Cav1-Y14D), were kind gifts from Dr. Nabi (University of British Columbia, Vancouver, BC, Canada). To generate Cav1-overexpressing clones, NMuMG cells were transfected with Lipofectamine 2000 (Invitrogen) and 4 μg DNA according to manufacturer’s instructions and selected by treatment with 800 μg/ml of G418 sulfate (Merck Biosciences) for 2 weeks. Single colonies were picked and checked for Cav1 protein levels by western blot analysis. To knockdown Cav1, small interfering (si)RNAs with four targeting sequences of 5′-GCUAUUGGCAAGAUAUUCA-3′, 5′-GCACAUCUGGGCGGUUGUA-3′, 5′-GCAAAUACGUGGACUCCGA-3′, and 5′-GUCCAUACCUUCUGCGAUC-3′ were used (SMART pool: ON-TARGET plus mouse Cav1 siRNA, Thermo Scientific, Rockford, IL, USA). Wild-type β1 integrin (WT-β1) and auto-clustering β1 integrin (V737N-β1) constructs were kindly provided by Dr. Weaver (University of California, San Francisco, CA, USA). Transient expression of Cav1 and β1 integrin were studied in HEK293T cells transfected with Lipofectamine 2000 (Invitrogen) according to the manufacturer’s instructions.

### Western blots analysis

Cell lysates were harvested with RIPA buffer containing additional 100 μM sodium orthovanadate (Na_3_VO_4_), 100 μM phenylmethanesulfomyl fluoride (PMSF), and protease inhibitor cocktail. The quantity of protein in each sample was quantified by Lowry assay. Protein lysates (30 μg) were resolved by SDS-PAGE and assessed by immunoblotting with specific primary antibodies against integrin β1 (clone18/CD29), Cav1, Cav1-pY14, FAK, FAK-pY397 (BD Biosciences PharMingen), talin, vinculin, and src (Santa Cruz). Protein levels of active integrin β1 were assessed by non-reducing gel with a specific antibody (clone 9EG7, BD Biosciences PharMingen)^30^.

### Immunofluorescence and image analysis

For immunocytochemistry, cells were fixed with 4% paraformaldehyde and permeabilized with 0.5% Triton X-100. Then, cells were incubated with blocking solution (Thermo Scientific) for 1 h and followed by incubation with specific primary antibodies. Antibodies against paxillin, β1 integrin, and active β1 integrin were purchased from BD Biosciences PharMingen. Antibodies against EEA1 were obtained from Abcam (Cambridge, UK). After washing with PBS, cells were incubated with secondary antibody for anti-mouse or rabbit IgG conjugated with fluorescent markers (Molecular Probes) and/or phalloidin-TRITC to label actin cytoskeleton (Fluka, Buchs, Switzerland) and 10 μg/ml Hoechst 33258 (Sigma-Aldrich) for 1 h. Lipid rafts were marked by cholera toxin subunit B (CTxB)-Alexa 488 (Molecular Probes). All images were taken with an FV-1000 confocal microscope (Olympus). The cell sizes, and focal adhesion (FA) sizes and numbers were quantified using Image Pro Plus software.

### Statistical analysis

All results are expressed as the mean ± SEM. Two-tailed Student’s t-test was used to compare differences between two groups, and the one-way analysis of variance (ANOVA) was used to compare differences when group numbers were greater than three. GraphPad Prism was used for the statistical analyses and statistical significance was set to p < 0.05.

## Electronic supplementary material

Supplementary information
