# Supplementary material for: Mechanotransduction of matrix stiffness in regulation of focal adhesion size and number: reciprocal regulation of caveolin-1 and β1 integrin
Source: Sci Rep. 2017 Nov 8;7:15008. doi: 10.1038/s41598-017-14932-6 (PMC5678369; doi:10.1038/s41598-017-14932-6)

Mechanotransduction of matrix stiffness in regulation of focal adhesion size and number: reciprocal regulation of caveolin-1 and β1 integrin

Yi-Chun Yeh, Jin-Ying Ling, Wan-Chun Chen, Hsi-Hui Lin*, and Ming-Jer Tang*

Department of Physiology, National Cheng Kung University, Tainan, Taiwan

*Corresponding Author:

Ming-Jer Tang, M.D., Ph.D.
Department of Physiology
National Cheng-Kung University Medical College, Tainan, Taiwan
Tel: 886-6-2353535 ext 5425
Fax: 886-6-2362780
E-mail: mjtang1@mail.ncku.edu.tw

*Co-Corresponding Author:

Hsi-Hui Lin, Ph.D.
Department of Physiology
National Cheng-Kung University Medical College, Tainan, Taiwan
Tel: 886-6-2353535 ext 5428
Fax: 886-6-2362780
E-mail: hsihuilin@mail.ncku.edu.tw

SUPPLEMENTARY MATERIAL INVENTORY

SUPPLEMENTARY FIGURES

Figure S1. The effects of CHX and low dose of trypsin on β1 integrin protein levels.

Figure S2. Disruption of lipid raft increase the endocytosis of β1 integrin.

Figure S3. Full-length western blots for Figures 2a, 2e, and 2f.

Figure S4. Full-length western blots for Figures 3c, and 3e.

Figure S5. Full-length western blots for Figures 4a, and 4b.

Figure S6. Full-length western blots for Figures 5a and 5d.

Figure S7. Full-length western blots for Figures 6a, 6c, 6e, and 6g.

Figure S8. Full-length western blots for Figure 7i.

Supplementary Figures

Figure S1. The effects of CHX and low dose of trypsin on β1 integrin protein levels. NMuMG cells grown on tissue culture dishes for two days were treated with or without 50 μg/ml cycloheximide (CHX) for 30min, cells were then suspended with low dose of trypsin (0.01% trypsin and 1mM EDTA). The cells lysates from each steps and different conditions as indicated were subjected to western blot analysis with an antibody against β1 integrin.

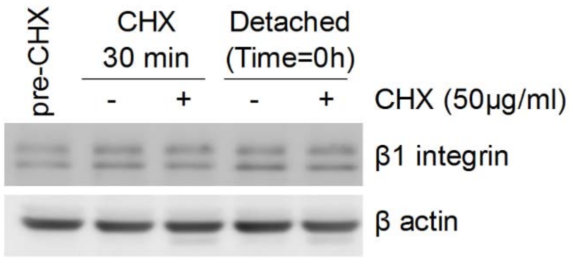

Figure S2. Disruption of lipid raft increase the endocytosis of β1 integrin. Confocal immunofluorescence images of NMuMG cells grown on the indicated conditions for 4 h. Cells were stained for β1 integrin (blue), active β1 integrin (green), and early endosome antigen 1 (EEA1) (red). Bar=10 μm.

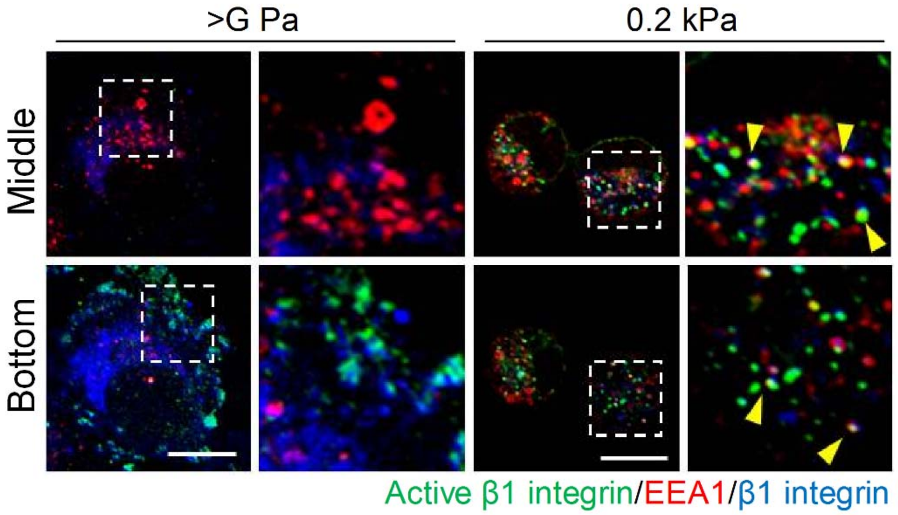

Figure S3. Full-length western blots for Figures 2a, 2c, and 2g.

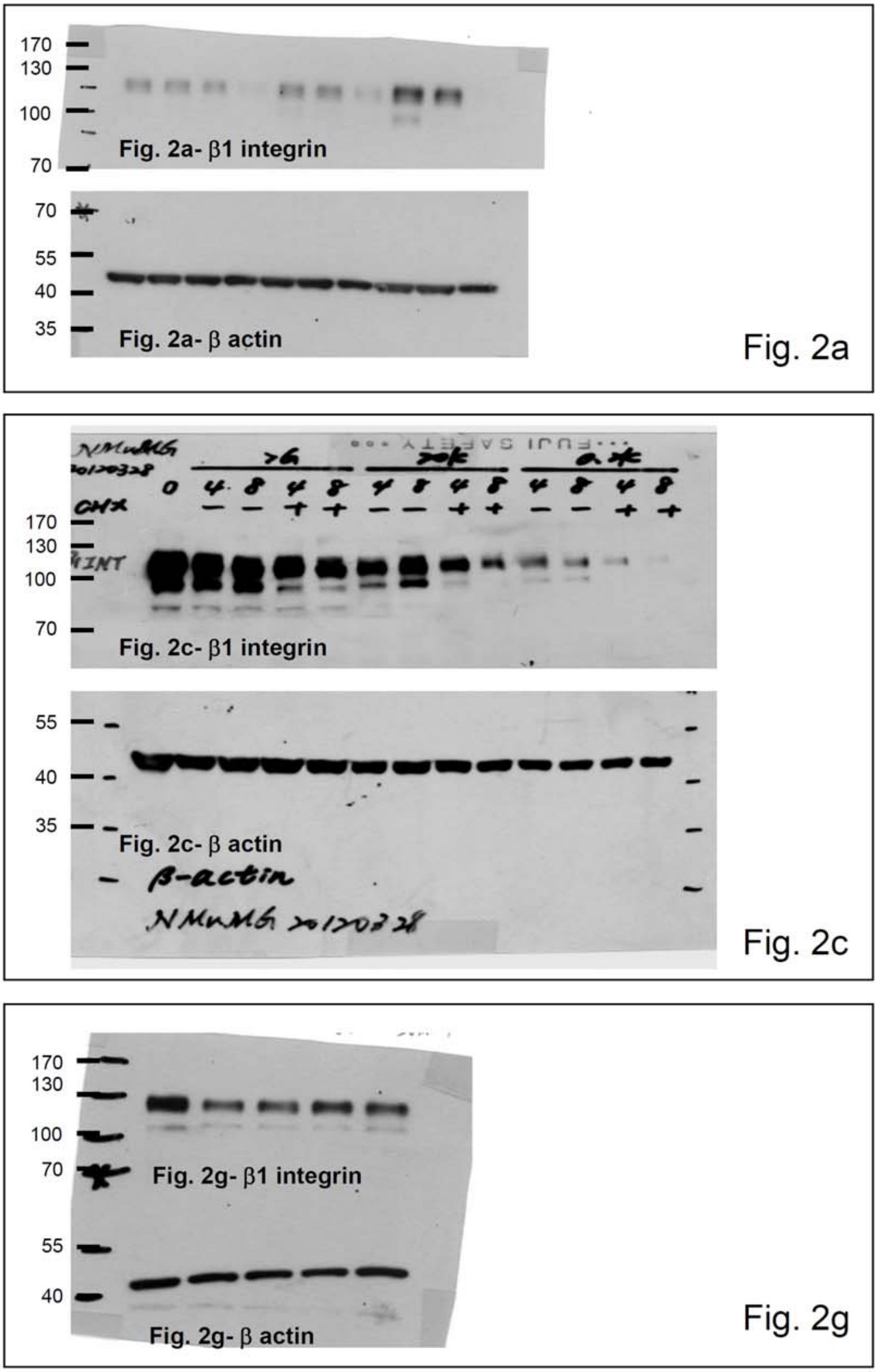

Figure S4. Full-length western blots for Figures 3c, and 3e.

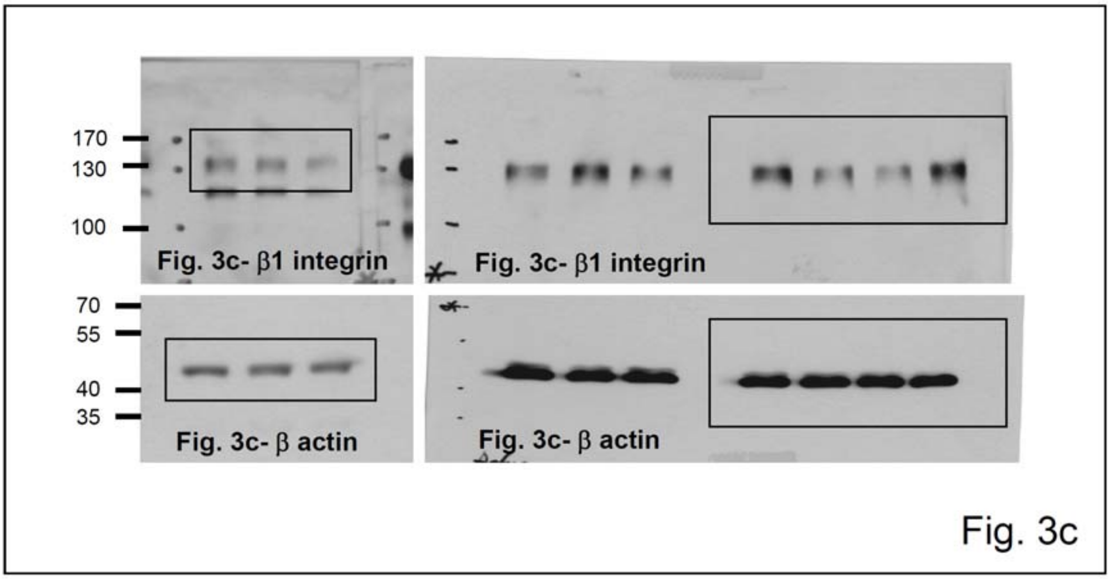

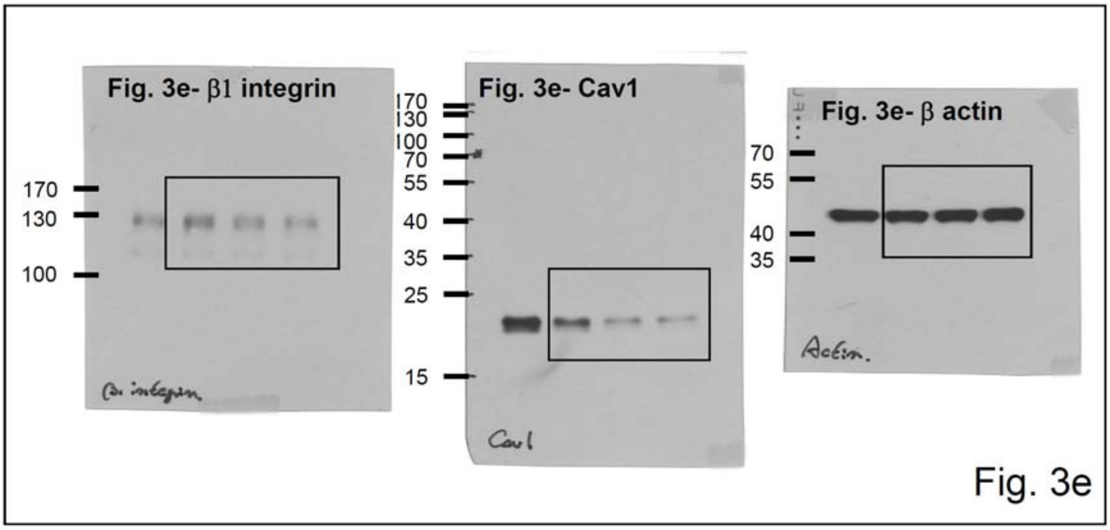

Figure S5. Full-length western blots for Figures 4a, and 4b.

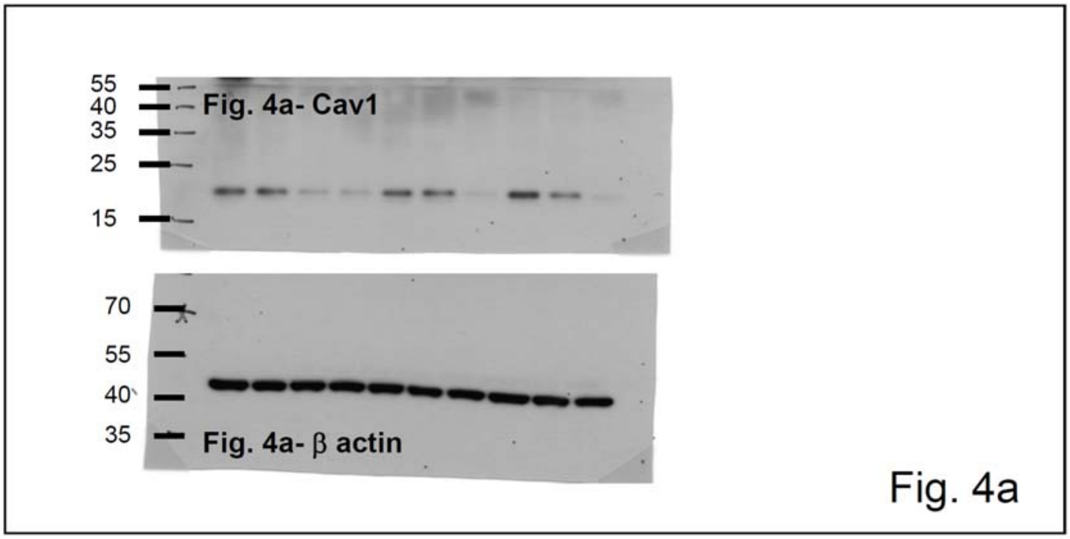

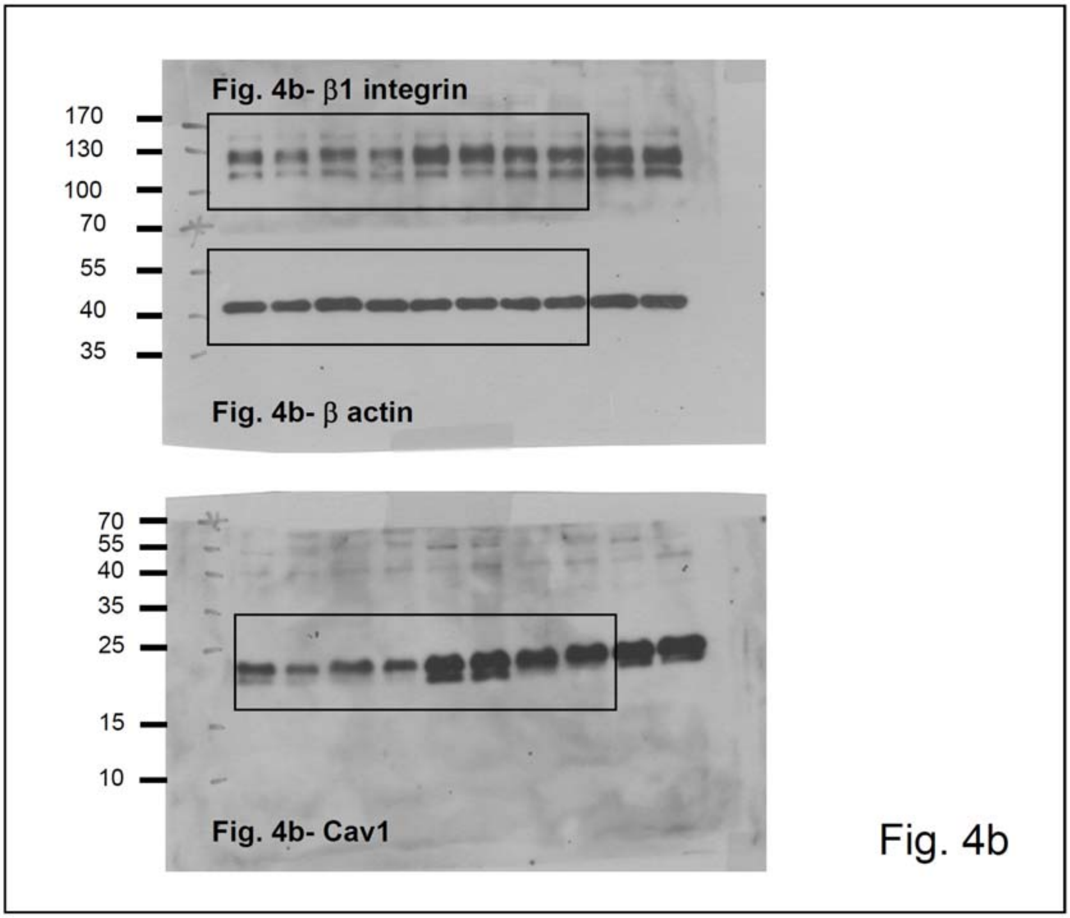

Figure S6. Full-length western blots for Figures 5a, and 5d.

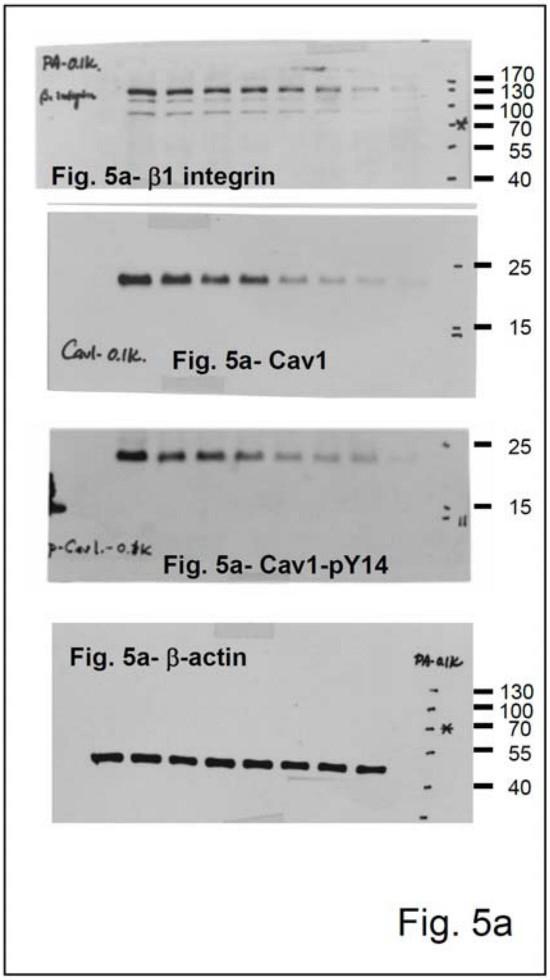

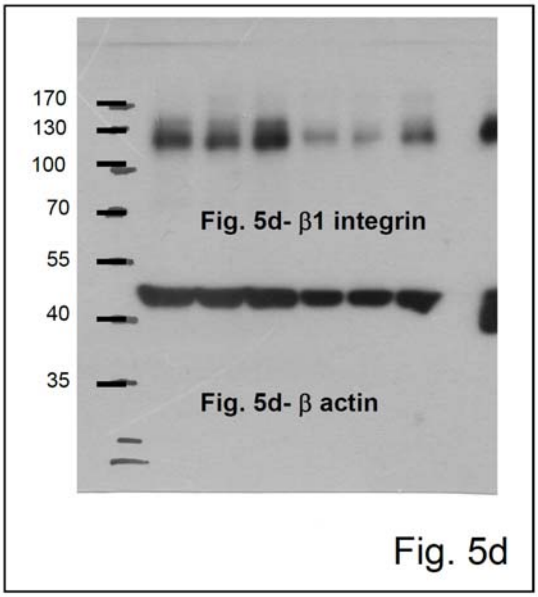

Figure S7. Full-length western blots for Figures 6a, 6c, 6e, and 6g.

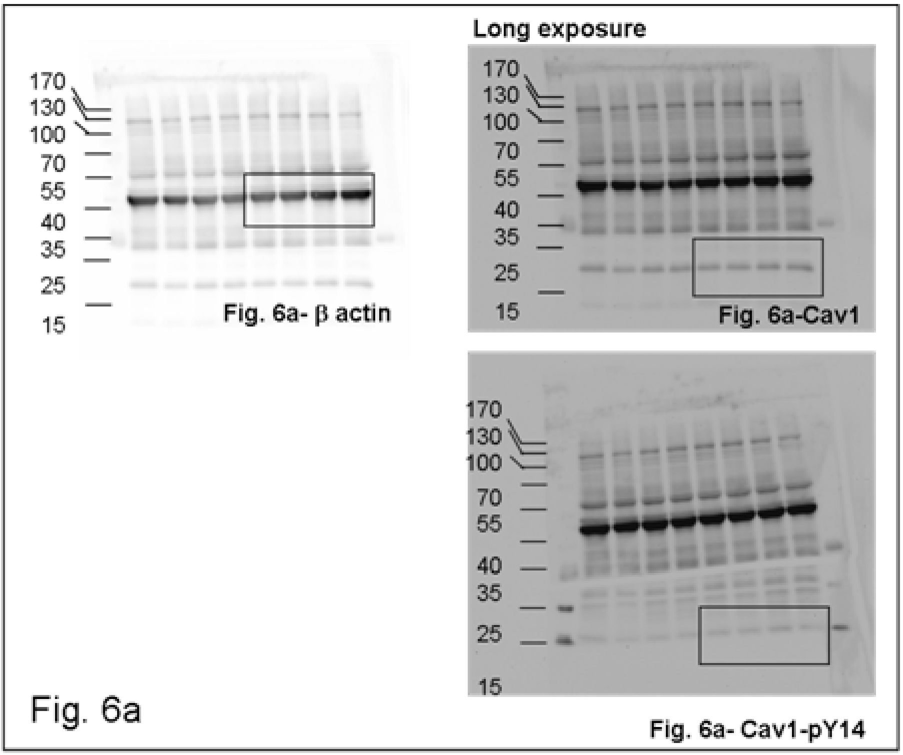

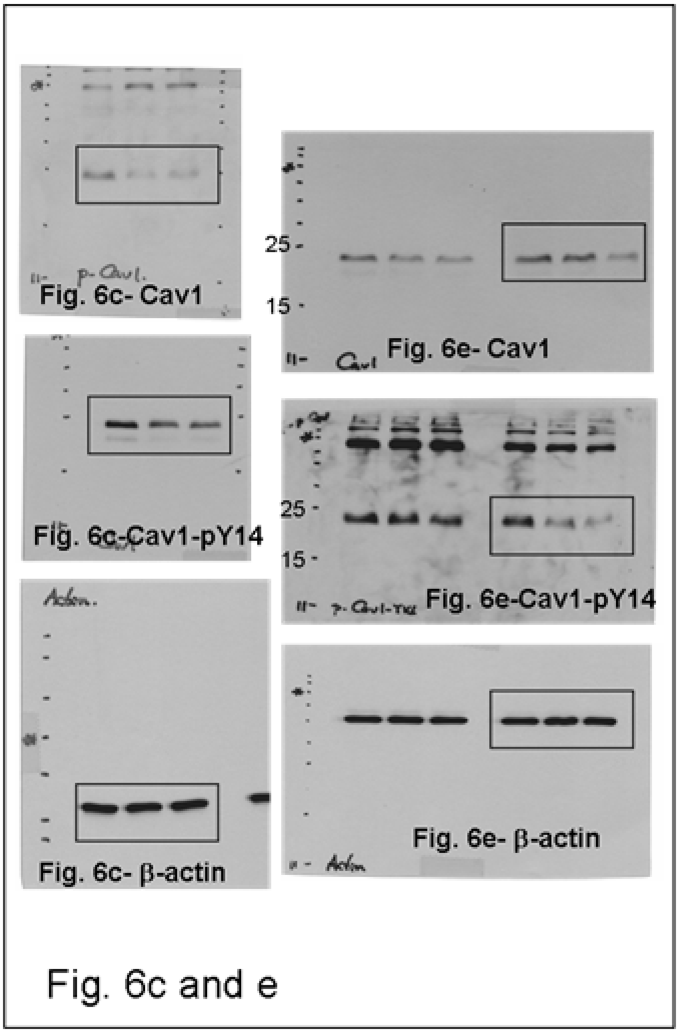

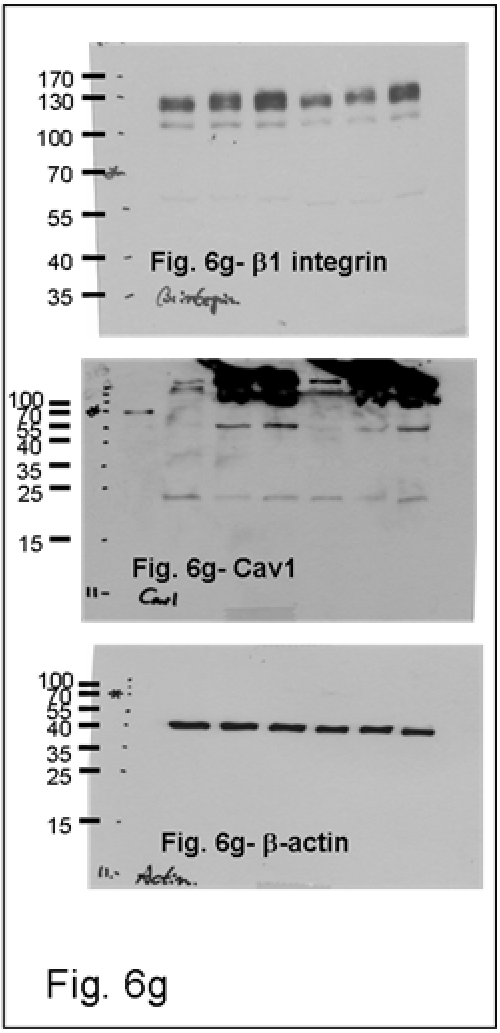

Figure S8. Full-length western blots for Figures 7i.

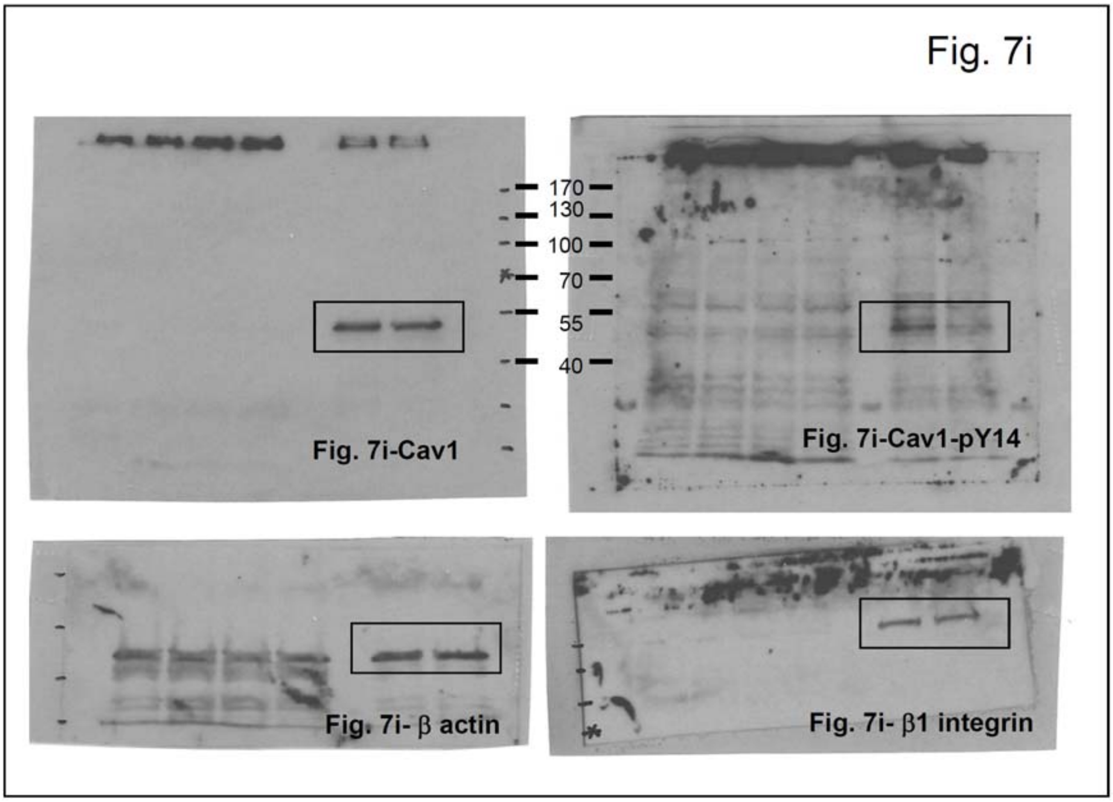

Supplement: Supplementary file 1 — Supplementary information [file 41598_2017_14932_MOESM1_ESM.pdf]
